# Unilateral Idiopathic Foveal Haemorrhage in a Young Female Adult

**DOI:** 10.1155/crop/5222043

**Published:** 2026-05-18

**Authors:** Michal Ruta, Marta Misiuk-Hojlo

**Affiliations:** ^1^ Clinical Department of Ophthalmology, 4th Military Clinical Hospital, Wroclaw, Poland; ^2^ Department of Ophthalmology, Wroclaw Medical University, Wroclaw, Poland, umed.wroc.pl

**Keywords:** fovea, haemorrhage, idiopathic, optical coherence tomography, retina

## Abstract

**Background:**

Unilateral idiopathic foveal haemorrhage is a rare condition, most often reported in otherwise healthy adults younger than 40 years, and is generally associated with a favourable prognosis.

**Case Presentation:**

We report the case of a 35‐year‐old woman who presented to the emergency department with isolated intraretinal foveal haemorrhage. Multimodal imaging, including optical coherence tomography (OCT), optical coherence tomography angiography (OCTA), fundus photography and follow‐up fluorescein angiography, was performed to assess the lesion localisation and exclude an underlying retinal vascular abnormality. Systemic evaluation did not identify any underlying cause.

**Conclusions:**

Foveal haemorrhage in a young, otherwise healthy patient is a diagnostic challenge and should be evaluated gradually, with a thorough medical history and multimodal imaging, to accurately diagnose and treat the patient. The treatment of idiopathic foveal haemorrhages has no standardised protocol and should be tailored to each case individually. Further research is needed to define evidence‐based management strategies for this patient group.

## 1. Background

Unilateral idiopathic foveal haemorrhage is a rare condition that may appear in otherwise healthy patients, usually younger than 40 years, with a reported female predominance and generally a favourable prognosis [1–3]. This manuscript presents a case report of a young female patient with isolated intraretinal foveal haemorrhage and discusses systemic and ophthalmological conditions that may underlie it. Our approach to the situation, diagnostic process and differential diagnosis is presented in the following sections. In the diagnostic phase, we focused on collaboration with other specialists and on multimodal imaging, which is crucial for the differential diagnosis of retinal diseases, including optical coherence tomography (OCT), optical coherence tomography angiography (OCTA) and fluorescein angiography. Since idiopathic foveal haemorrhage is relatively uncommon, the diagnostic challenge it poses, the differential diagnostic process and the subsequent management strategy are directly applicable to routine clinical practice. The sequential exclusion of systemic causes and the clinical decision‐making processes involved may offer valuable insights for ophthalmologists confronted with presentations of a similar, initially ambiguous nature. The key to the diagnostic process and treatment lies in collaboration with other specialists, such as primary care physicians, cardiologists, haematologists and oncologists.

## 2. Case Presentation

**Figure 1:**
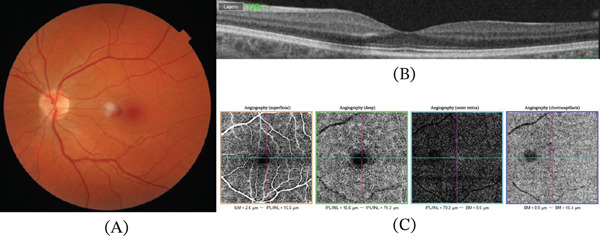
Multimodal imaging of the patient at presentation. (A) Fundus photography with a visible intraretinal haemorrhage at the fovea. (B) OCT with a visible intraretinal hyperreflective signal in the outer plexiform layer. (C) OCTA.

**Figure 2:**
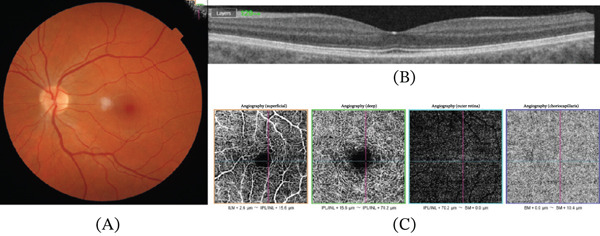
Follow‐up visit documentation. (A) Colour photograph of the fundus showing no visible signs after the haemorrhage. (B) OCT revealing barely visible deterioration in the outer plexiform layer. (C) OCTA.

**Figure 3:**
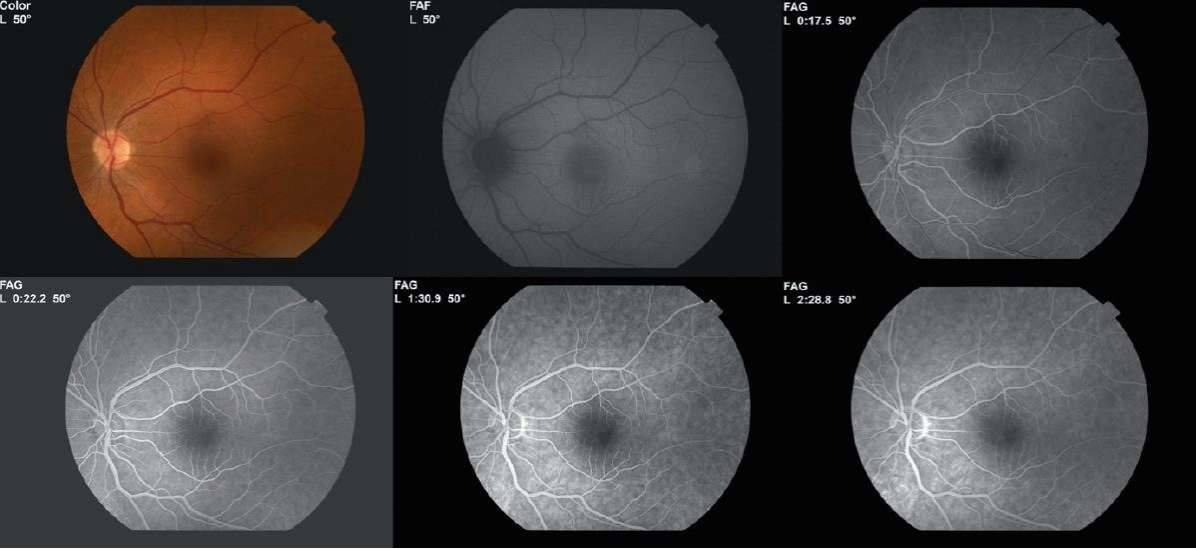
Angiography revealed normal vasculature at the follow‐up visit.

A 35‐year‐old female patient was admitted to our emergency department with a complaint of visual distortion in the left eye, characterised by scotomas and intermittent flashes in the visual field. She denied ocular trauma, high myopia, recent strenuous physical activity or Valsalva manoeuvres (including coughing, straining, vomiting or heavy lifting), medication use, including anticoagulant or antiplatelet therapy, and known systemic disease. The patient was not pregnant, nor in the postpartum period, and no abnormal menstrual bleeding had been reported. The best‐corrected visual acuity (BCVA) in the left eye was 1.0 (Snellen chart), read with difficulty and requiring a change in head position, whilst the BCVA in the right eye was 1.0 (Snellen chart). The anterior segment and intraocular pressure were regular. A diminutive, solitary, flat, regular, dot‐shaped intraretinal haemorrhage was identified in the left eye, as shown in the fundus photo (Figure 1A), which corresponded with a hyperreflective signal in the outer plexiform layer in the OCT (Figure 1B). The vitreoretinal interface was intact, with no tractional abnormality. This localisation is consistent with haemorrhages arising from the deeper retinal layers, which typically manifest as dot‐ or blot‐shaped lesions on fundus examination. There were no exudates or accompanying visible vascular anomalies around this haemorrhage. The OCTA scans (Figure 1C) also did not show any pathological signs, such as neovascularisation or abnormal vascularisation in the macula. Exceptionally, fluorescein angiography was deferred due to organisational constraints, in the context of a negative OCTA examination and maintained visual acuity. The patient received an intravitreal injection of anti‐VEGF 2 mg aflibercept. Afterwards, the patient was referred to the general practitioner for further investigation of possible systemic illnesses. At the 4‐week follow‐up, the haemorrhage was no longer clinically visible (Figure 2A). The place after the haemorrhage was only recognised on OCT as a slight irregularity in the outer plexiform layer (Figure 2B). No pathological signs were observed in OCTA (Figure 2C). The visual acuity of the left eye was 1.0, read without difficulty and without requiring a change in head position. The patient reported only a slight deterioration in the visual field. The examination was extended, and fluorescein angiography (Figure 3) was performed. No abnormalities that could underlie the treated condition were identified. The complete blood count, routine serum biochemical tests, coagulation function tests and cardiological and haematological consultations did not reveal any underlying cause. During the 8‐month periodic ophthalmological follow‐up, no recurrence or new clinically relevant findings were observed.

## 3. Discussion

The patient with retinal haemorrhage is a medical challenge. Several conditions, such as retinal or systemic diseases, blunt trauma, thoracic trauma and increased intracranial pressure, should be considered in the diagnostic process to provide an appropriate therapeutic strategy.

The first issue to address is the location of the haemorrhage—preretinal, intraretinal or subretinal. The intraretinal haemorrhages can be flame‐shaped or dot‐ and blot‐shaped. The first ones are located superficially, primarily at the posterior pole, and have irregular, feathery borders. The other group comprises haemorrhages located in the deep retinal layers, with a regular, dot‐ or blot‐like appearance on fundoscopy, as in our patient’s case. The haemorrhages at the periphery of the retina are commonly dot‐ and blot‐shaped, regardless of the location. The subretinal haemorrhages are located deeper than the retinal vessels [4, 5]. The preretinal group may assume a boat‐shaped configuration and fill the vitreous cavity.

The next issue is whether involvement is limited to one or both eyes. The most frequent cases of unilateral haemorrhages are due to retinal vein occlusion. On the other hand, bilateral cases are more complex and are generally associated with systemic diseases.

Furthermore, the location of the haemorrhage must be observed. A restriction to the posterior pole may suggest systemic involvement, whereas extension to the periphery is primarily observed in venous occlusive disease.

Diabetic retinopathy is typically bilateral and affects small retinal vessels, leading to increased vascular permeability, retinal haemorrhages, microaneurysms, cotton‐wool spots, exudates, retinal oedema, ischaemic areas and neovascularisation.

In hypertensive retinopathy, changes occur in the arterial network, starting with arterial narrowing and arterial sclerosis, progressing to wall opacification, which may then advance to later stages with haemorrhages, optic disc swelling, microaneurysms and exudates.

Valsalva retinopathy is an uncommon disorder observed in younger patients, resulting from sudden increases in ocular venous pressure, for example, during weight lifting, childbirth, balloon blowing or intensive physical exercise. Haemorrhages may present as pre‐, intra‐ or subretinal. Occasionally, the subretinal limiting membrane haemorrhage may break into the vitreous cavity or subhyaloid space [6].

One‐third of patients with anaemia will present signs of retinopathy, especially with coexisting thrombocytopenia. It may manifest as superficial haemorrhages, cotton‐wool spots and retinal oedema. It is less common to identify a macular star or preretinal and vitreous haemorrhages in this group of patients. The haemorrhages are mainly superficial and flame‐shaped; they may be white in the centre. What is more, venous tortuosity may be present during fundus examination and increased retinal transit time in fluorescein angiography. In most cases, visual acuity is intact, except in patients with macular involvement. The presence of anaemia may exaggerate diabetic and leukaemic retinopathy. It remains unclear which level of anaemia or haemoglobin concentration is associated with retinopathy. It is probably influenced by age, gender and comorbidities. The presence of the signs listed above is suggestive of anaemia but not diagnostic; therefore, it is crucial to perform a routine blood test to confirm the diagnosis [5, 7, 8].

Fifty percent of patients with leukaemia may present with retinal symptoms. The initial manifestation may be retinal vein occlusion. Symptoms such as flame‐shaped intraretinal haemorrhages, dilated and tortuous veins, microaneurysms, cotton‐wool spots, vessel sheathing and white masses in the retina or optic disc are characteristic of leukaemic retinopathy. It is more likely to detect retinopathy symptoms in adult patients and in cases of acute and myelogenous leukaemia. Furthermore, the clinical presentation in leukaemia may be influenced by coexisting anaemia, thrombocytopenia and hyperviscosity [5, 9–11].

The retinopathy associated with polycythaemia typically manifests as dilated, tortuous, dark veins, a hyperaemic, swollen optic disc and intraretinal haemorrhages. It could also manifest as central or branch retinal vein occlusion. The symptoms are mainly bilateral. There are also published cases in which polycythaemia has led to central retinal artery occlusion, peripheral neovascularisation and macular haemorrhages [12–14]. The clinical signs of polycythaemia are significant when the haematocrit exceeds 50%.

In thrombophilias, clinical manifestations may include thrombotic events, such as central and branch retinal vein occlusion or nonarteritic anterior ischaemic optic neuropathy [5].

Idiopathic macular telangiectasia (MacTel) Type 1 is a developmental or congenital retinal vascular disease that typically occurs unilaterally in males. It can be diagnosed at any age, but it is most often confirmed at the age of 40. It is characterised by capillary ectasia and dilatation in the juxtafoveal region, which leads to vascular leakage, haemorrhages, macular oedema and lipid deposition [1, 15].

Exposure to any radiation may result in radiation retinopathy. Microaneurysms, telangiectasias, hard exudates and haemorrhages, macular oedema, neovascularisation and tractional retinal detachment are manifestations of it.

Purtscher’s retinopathy may occur due to recent major trauma, pancreatitis, childbirth or renal failure. The patient presents with ischaemia at the posterior pole, oedema and haemorrhages around the optic disc.

Possible treatments for idiopathic foveal intraretinal haemorrhages include observation, anti‐VEGF treatment, tissue plasminogen activator–assisted clot lysis, pneumatic displacement or surgical management. The final visual outcomes are closely linked to the background of haemorrhages and retinal changes due to blood‐iron toxicity, fibrin concentration and scar formation [4]. Although in our patient’s case OCTA and fluorescein angiography did not reveal neovascularisation, active leakage or abnormal vascular structures, anti‐VEGF therapy may exert beneficial effects by reducing subclinical vascular permeability and inflammatory activity, thereby facilitating haemorrhage resorption and promoting retinal recovery. Our consideration of intravitreal anti‐VEGF injection was based on several publications on the mechanisms and effects in various retinal diseases. It was published that anti‐VEGF therapy significantly improves visual acuity in patients with severe macular haemorrhage [16]. Moreover, it is known that anti‐VEGF injections may help reduce vitreous haemorrhage. Anti‐VEGF is also helpful in the treatment of macular oedema in retinal vein occlusion by antagonising VEGF and/or placental growth factor (PGF), thereby reducing inflammation and retinal hypoxia [17]. There are also publications concerning the successful treatment of preretinal haemorrhage in Valsalva retinopathy [18, 19].

The spontaneous resolution of small idiopathic intraretinal haemorrhages in young patients is a plausible outcome; therefore, it is not possible to definitively determine whether the resolution observed in this case was a direct result of anti‐VEGF therapy or part of the natural course of the condition. Since there is no standardised treatment protocol for idiopathic foveal haemorrhages and to minimise the potential adverse effects of prolonged intraretinal blood persistence, following a shared decision‐making process with the patient, we elected to proceed with intravitreal anti‐VEGF therapy.

## 4. Conclusions

The foveal haemorrhage may occur in young, otherwise healthy patients without any predisposing factors. However, it is crucial to rule out systemic or retinal conditions as underlying causes. The patient’s diagnosis should always include clinical manifestations, medical history, fundoscopy, visual acuity assessment, intraocular pressure measurement and multimodal imaging. Ophthalmologists should be aware that various manifestations of retinopathy may indicate a systemic disorder. In these cases, the cooperation of ophthalmologists, primary care physicians, cardiologists, haematologists and oncologists is crucial throughout the diagnostic process and treatment. Idiopathic macular haemorrhage is typically diagnosed in women younger than 40 years with healthy eyes. The etiopathogenesis is still unknown. Treatment in these cases should be tailored to each case, as there is no standardised protocol for achieving the best possible outcome. In selected patients with preserved vision and no evidence of neovascularisation or leakage, observation should be considered, whereas interventional treatment should be justified by the clinical context and discussed with the patient. Further research on idiopathic haemorrhages is crucial to achieve a protocol for treatment in this group of patients.

**Nomenclature**
OCT: optical coherence tomography
OCTA: optical coherence tomography angiography
BCVA: best‐corrected visual acuity
MacTel: macular telangiectasia
PGF: placental growth factor

## Author Contributions

Both authors contributed equally to the preparation, writing and revision of the manuscript.

## Funding

No funding was received for this manuscript.

## Disclosure

Both authors read and approved the final version.

## Ethics Statement

The authors have nothing to report.

## Consent

Written informed consent has been obtained from the patient to publish this paper.

## Conflicts of Interest

The authors declare no conflicts of interest.

## Data Availability

Data sharing is not applicable.
